# Neuronal biomarkers and hippocampal volume in childhood-onset systemic lupus erythematosus

**DOI:** 10.1186/1546-0096-12-S1-P319

**Published:** 2014-09-17

**Authors:** Aline Lapa, Renata Barbosa, Mariana Postal, Nailu Sinicato, Fernando Cendes, Roberto Marini, Simone Appenzeller

**Affiliations:** 1 Medicine, State University of Campinas, Campinas, Brazil; 2 Neurology, State University of Campinas, Campinas, Brazil; 3 Pediatrics, State University of Campinas, Campinas, Brazil

## Introduction

Hippocampal atrophy is associated with corticosteroid use and may be related to cognitive impairment in systemic lupus erythematosus (SLE). Some biomarkers associated with neuronal injury have been associated with neuropsychiatric SLE, but their roles in the pathogenesis and its validity and clinical applicability has not been studied in childhood-onset systemic lupus erythematosus (cSLE).

## Objectives

To determine the possible relationship between hippocampal volume loss and S100β e subunit of high molecular weight neurofilament (NF-H).

## Methods

We included consecutive cSLE patients followed in a cohort at the pediatric rheumatology unit at the State University of Campinas and age and sex matched healthy controls. All patients had disease-onset before the age of 18. Magnetic resonance imaging (MRI) scans were obtained through a standardized protocol (3Tessla Philips). Volumetric 1mm Tl weighted images were used for manual volumetric measurements. Volumes smaller 2 standard deviation from the means of controls were considered abnormal. Non-parametric-tests and correlation were used for statistical analysis. S100β and NF-H protein levels were measured by enzyme-linked immunosorbent assay using commercial kits from BioVendor, Inc (Czech Republic) and compared by non-parametric tests.

## Results

We included 71 cSLE patients (64 female; mean age 18.8 ±4.06 years) and 50 healthy controls with similar age and sex distribution. cSLE had a mean disease duration of 6.38 ±4.54 years. Neuropsychiatric manifestations were observed in 49 (69.0%) patients. The volumes of right (mean volume 3.38±0.57 cm^3^) and left (mean volume 3.40±0.60 cm^3^) hippocampi were significantly smaller when compared to controls (right: mean volume 4.4±0.5 cm^3^, p < 0.001; left: mean volume 4.43±0.45 cm^3^, p <0.001). Hippocampal atrophy was identified in 46 (64.80%) patients. NF-H protein levels were increased in cSLE (101.80±89. 40 pg/mL) when compared to controls (57.12±13.28 pg/mL; p=0.038). Serum S100β levels were significantly increased in cSLE (148.98 ± 102.73 pg / mL) compared to controls (48.10 ± 38.52 pg / mL; p <0.001). No association of S100B and NF-H levels in cSLE patients with and without neuropsychiatric manifestations was observed. No association between S100B and NF-H with hippocampal volumes or hippocampal atrophy was observed.

## Conclusion

Neuropsychiatric manifestations and hippocampal atrophy is frequently observed in cSLE patients, however no association with neuronal biomarkers was observed. Further studies need to be done to determine biomarkers for hippocampal involvement in cSLE.

## Disclosure of interest

A. Lapa: None declared, R. Barbosa: None declared, M. Postal: None declared, N. Sinicato: None declared, F. Cendes: None declared, R. Marini: None declared, S. Appenzeller Grant / Research Support from: FAPESP (2008/02917-0, 2011/03788-2, 2013/09480-5) CNPQ (300447/2009-4 and 471343/2011-0; 302205/2012-8; 473328/2013-5)

